# A View on the Role of Epigenetics in the Biology of Malaria Parasites

**DOI:** 10.1371/journal.ppat.1002943

**Published:** 2012-12-13

**Authors:** Alfred Cortés, Valerie M. Crowley, Alejandro Vaquero, Till S. Voss

**Affiliations:** 1 Barcelona Centre for International Health Research (CRESIB, Hospital Clínic-Universitat de Barcelona), Barcelona, Catalonia, Spain; 2 Catalan Institution for Research and Advanced Studies (ICREA), Barcelona, Catalonia, Spain; 3 Institute for Research in Biomedicine (IRB), Barcelona, Catalonia, Spain; 4 Cancer Epigenetics and Biology Program (PEBC), Bellvitge Biomedical Research Institute (IDIBELL), L'Hospitalet de Llobregat, Barcelona, Catalonia, Spain; 5 Department of Medical Parasitology and Infection Biology, Swiss Tropical and Public Health Institute, Basel, Switzerland; 6 University of Basel, Basel, Switzerland; The Fox Chase Cancer Center, United States of America

Cells and unicellular organisms are similar to their progenitors because information is transmitted from one generation to the next. The information is mainly transmitted in the primary sequence of the genome (genetic information), but there are heritable traits that are transmitted by other mechanisms. Epigenetics studies these alternative modes of inheritance. According to classic definitions, epigenetics refers to heritable differences between cells or organisms that occur without changes in DNA sequence, and do not depend on different external conditions [1]–[4].

Epigenetic information can be transmitted by several different molecular mechanisms, which include but are not limited to DNA methylation and histone post-translational modifications (PTMs). However, the term “epigenetics” is often used to refer to any transcriptional regulation mechanism that involves histone PTMs or other chromatin-based processes, and the study of chromatin modifications on a genome-wide level is commonly termed “epigenomics”. Several voices have questioned this use of the word epigenetics, because many histone PTMs do not carry heritable information [1]–[8]. A closely related debate around chromatin modifications is about causality: several histone PTMs correlate with specific transcriptional states, but in many cases they are not responsible for a transcriptional outcome but rather are a consequence of it [7]. Hence, the debate about the use of the term epigenetics is a terminology issue that affects our understanding of how cellular processes are ultimately controlled. In some processes, chromatin modifications carry heritable regulatory information that is transmitted from mother to daughter cells, whereas in other cases, they are implicated in the execution of the information contained in the DNA sequence, or occur as a consequence of dynamic nuclear processes such as transcription.

In the last few years, chromatin modifications have been extensively studied in the malaria parasite *Plasmodium falciparum* (for recent reviews, see [9]–[13]). Many processes in parasite biology involve changes at the chromatin level, including regulation of transcription along a complex life cycle, delimitation of functional elements in the genome, and antigenic variation. Here we will describe our current knowledge of the biological processes and mechanisms that can be considered bona fide epigenetic phenomena in *Plasmodium* biology, and attempt to distinguish them from those unlikely to involve epigenetic flow of information, even if chromatin changes occur. We will not judge the use of the term epigenetic in different situations, but will rather attempt to clarify the roles of chromatin-based modifications in the different processes.

## Epigenetic Processes in Malaria Parasite Biology

### Variant Gene Expression

**Figure 1:**
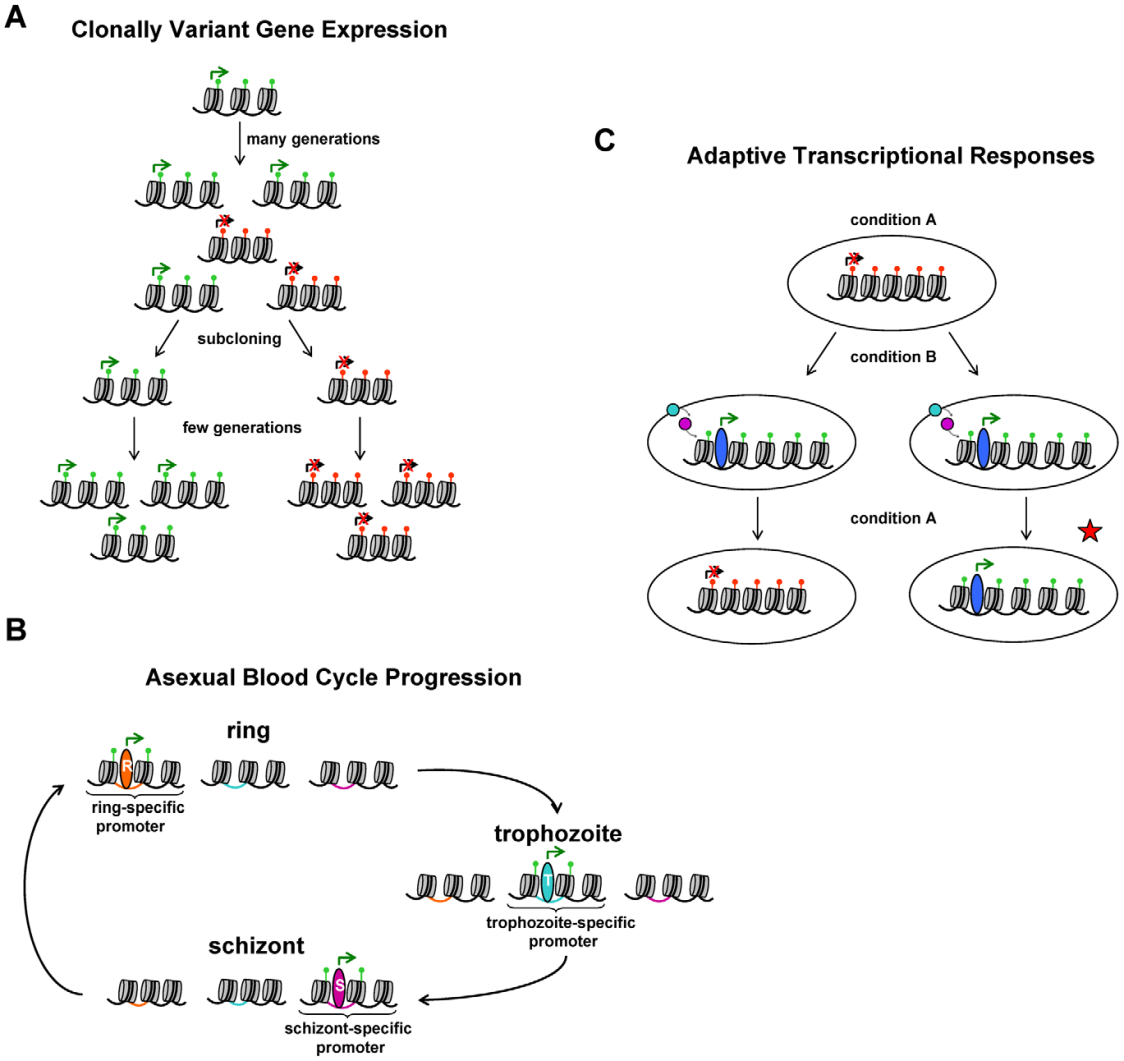
Schematic representation of processes in parasite biology that involve chromatin changes. (**A**) CVGE. Expression dynamics of a representative clonally variant gene. In a clonal population, stochastic switches between the active and silenced states of the gene result in transcriptional heterogeneity upon long-term growth. The active state is associated with activating histone PTMs (e.g., H3K9ac, green marks), whereas the silenced state is associated with repressive histone PTMs (e.g., H3K9me3, red marks). Subcloning followed by short-term growth results in populations of parasites that predominantly maintain the same transcriptional state as the single parasite from which they are derived, demonstrating epigenetic inheritance. (**B**) Asexual blood cycle progression. The three stretches of chromatin represent a gene expressed only in ring stages, trophozoites, or schizonts, respectively. Hypothetical stage-specific transcription factors (represented by ovals) control expression of the genes by acting on regulatory elements in the promoter sequences (colored DNA). Some histone PTMs are associated with active transcription (green marks), and global changes in chromatin organization occur at specific stages (e.g., higher nucleosome density in schizonts). It is unlikely that these modifications are stably transmitted from one generation to the next because they change during each cycle. (**C**) Adaptation via directed transcriptional responses. A change in the environment is sensed and via signal transduction (colored circles) results in a directed transcriptional response, which can operate via changes in chromatin structure (e.g., deposition of activating histone PTMs). Only if the chromatin changes and transcriptional status are maintained after the external signal disappears, is there epigenetic transmission of information (red star). To date, there is no well-described pathway in *P. falciparum* that involves sensing external conditions followed by a directed transcriptional response, but here we propose a conceptual framework to determine whether there is epigenetic inheritance of transcriptional states if such a pathway is ever identified.

Clonally variant gene expression (CVGE) lies at the base of a bet-hedging adaptive strategy consisting of the stochastic generation of phenotypic diversity followed by natural selection upon environmental changes (for a recent discussion on bet-hedging, see [14]). The genome of *P. falciparum* contains hundreds of genes that show CVGE, such that individual parasites within an isogenic population express these genes at very different levels, often fully active or completely silenced [15]. The transcriptional patterns in each parasite are clonally transmitted over multiple generations of asexual growth, with stochastic switches between the active and silenced states occurring at low frequency (Figure 1A). In *P. falciparum*, many clonally variant genes belong to large and mostly subtelomeric multigene families that are involved in antigenic variation [9], [15]–[17]. The best characterized gene family showing CVGE is the *var* gene family, which consists of about 60 genes per genome encoding the red blood cell surface antigen *P. falciparum* erythrocyte membrane protein 1 (PfEMP-1) [18]–[21]. In this and other cases, the most likely function of CVGE is immune evasion. However, recent work has demonstrated that CVGE in *P. falciparum* also occurs in many other gene families linked to different processes such as erythrocyte invasion, nutrient transport, protein folding, and lipid metabolism [15], [22]–[25].

CVGE in *P. falciparum* fulfills even the most stringent definitions of epigenetics: two parasites with identical genomes growing under identical external conditions (even in the same culture dish) can maintain a variant gene (e.g., a *var* gene) in a different transcriptional state, active or silenced, and this state will be transmitted to the next generations (with infrequent switches). This has been clearly demonstrated by subcloning experiments (Figure 1A) [15], [16], [22], [26]. In some cases, epigenetic changes may be triggered by an external signal (sometimes referred to as an “epigenator” [3]), such as environmental factors or developmental cues, and then maintained in the absence of the signal [4], [27]. However, there is no evidence to date for the involvement of external signals in CVGE in *P. falciparum* [28]. Unknown external triggers may play a role in inducing CVGE, but the observation that changes in expression occur spontaneously during normal growth in parasite cultures suggest that epigenetic changes occur stochastically as a consequence of intrinsic factors. In this regard, transmission of epigenetic marks is less faithful than DNA replication, which offers an opportunity for transitions between chromatin states [8]. An intrinsic propensity to stochastic transitions in the chromatin domains where clonally variant genes are located may lie at the basis of CVGE.

### Molecular Mechanisms Controlling CVGE

Genome-wide chromatin analysis revealed a remarkable correlation between the distribution of heterochromatin marks and clonally variant genes [15], [29]–[31], indicating that the formation of heterochromatin is a general mechanism controlling CVGE in *P. falciparum*. Studies on specific gene families, including *var* and others, also support this view [32]–[37]. *P. falciparum* clonally variant genes are located in bistable chromatin domains [36], which can be found in two alternative conformations, permissive (euchromatin) or repressive (heterochromatin). Once established, both chromatin conformations are stably maintained over multiple generations (see [38] for a theoretical framework of bistable chromatin domains). The active state of clonally variant genes from different gene families is associated with permissive histone marks, including acetylation at histone H3 lysine 9 (H3K9ac) and di- or tri-methylation at H3K4 (H3K4me2/3). On the other hand, the silenced state of clonally variant genes is characterized by H3K9me3, a repressive mark characteristic of heterochromatin [32]–[36]. Importantly, H3K9 PTMs, and in *var* genes also H3K4 PTMs, are maintained through stages of the asexual blood cycle at which the genes are not expressed, indicating that they have the potential to constitute the epigenetic mechanism that transmits cellular memory [32]–[34], [36]. Persistence of a chromatin mark throughout the cell cycle is a prerequisite for it to act as a heritable/epigenetic mark. Of note, H3K9me3 is among the few histone PTMs for which a plausible self-perpetuating mechanism of transmission through cell division has been proposed, analogous to the positive feedback loops used for the spreading of chromatin states into neighbor regions. For most other histone PTMs, it remains to be determined whether they can be maintained throughout DNA replication [6], [8], [39]. H3K9me3 is an evolutionarily conserved histone PTM typically mediating chromatin compaction [40]; its role in controlling reversible silencing of clonally variant genes predicts an important role for H3K9me3 demethylases in *P. falciparum*. In this regard, a demethylase with possible specificity for this mark has been identified in the parasite's genome [41]. It is possible that other histone PTMs involved in silencing in other eukaryotes, such as H4K20me3 or H3K27me3, may contribute to epigenetic silencing in *P. falciparum*. However, H4K20me3 shows a broad distribution in the genome that does not correlate with clonally variant genes [30], and H3K27me3, which is involved in cell type–specific epigenetic silencing in multicellular eukaryotes, has not been identified to date in *P. falciparum* [42].

The molecular players involved in regulating histone PTM patterns in CVGE have been partially characterized only for *var* genes. The *var* gene promoter and the intron, the ApiAP2 DNA-binding protein PfSIP2, the two histone deacetylases PfSIR2A and PfSIR2B, the H3K4 methyltransferase PfSET10, heterochromatin protein 1 (PfHP1), and the histone variant H2A.Z all contribute to specify the active or silenced state of a *var* gene [26], [29], [43]–[51]. However, it is still unclear which of these factors are involved in the transmission of epigenetic information and which are downstream effectors in the process. DNA methylation is apparently absent in *P. falciparum* [52], and the same is true for the RNA interference machinery [53]. Non-coding RNAs (ncRNAs), which are common players in epigenetic regulation in other organisms [54], are abundant in *P. falciparum*, and conceivably they may play a role in the control of CVGE [55]–[59]. Antisense transcripts are particularly common, and long ncRNAs encoded in subtelomeric regions, where most clonally variant genes are located, have recently been identified [60], [61]. Intriguingly, these long ncRNAs originate from regions containing abundant SPE2 motifs [60]. These *cis*-acting DNA elements are bound by the ApiAP2 factor PfSIP2, and this interaction plays a likely role in *var* gene regulation [49]. In the case of *var* genes, ncRNAs initiated from *var* introns are involved in the control of a mutually exclusive expression program [57], but the functional role of other *Plasmodium* ncRNAs still awaits experimental characterization. Another important layer of CVGE regulation is subnuclear localization. Activation of a *var* gene is associated with spatial repositioning within the nucleus to a specific perinuclear active site, possibly involving dissociation from telomeric clusters [30], [44], [46], [62], [63], in a process that involves the *var* intron and actin [64]. Whether changes in subnuclear localization are also involved in activation of other clonally variant genes remains to be demonstrated. Considering that *Plasmodium* parasites undergo closed mitosis and that their chromosomes do not condensate during nuclear division [65], it is conceivable that the localization of a clonally variant gene within the nucleus may be maintained through nuclear division and consequently transmitted from one generation to the next, thus contributing to epigenetic inheritance of the expression status. Interplay between histone PTMs and subnuclear localization, such that one determines or reinforces the other, has been observed in other organisms [66].

## Other Chromatin-Based Processes in Malaria Parasites

### Life Cycle Progression

Histone PTMs and other chromatin modifications are key regulators of transcription in eukaryotes [67]. In addition to CVGE, several other processes in *Plasmodium* biology are regulated at the transcriptional level and are associated with changes in chromatin structure. A key process that is largely controlled at the transcriptional level is the progression along the life cycle, which involves multiple stages in two different hosts, the *Anopheles* mosquito and humans [68]. The majority of genes are expressed only at stages of the life cycle when their products are needed, and are repressed during the rest of the life cycle [69], [70], a pattern that is often referred to as a “just in time” type of expression. Hourly microarray analysis during intra-erythrocytic development has revealed a continuous cascade of gene expression as the parasite progresses through the asexual blood cycle [69]. This cascade of gene expression is accompanied by global fluctuations in nucleosome density and differential distribution of certain histone PTMs [31], [71]–[73]. Interestingly, H3K9ac occupancy is positively correlated with the temporal pattern of gene transcription, whereas H3K4me3 levels generally increase in trophozoites and schizonts, largely independent of temporal gene activity [72]. Further evidence for the involvement of histone PTMs in *P. falciparum* asexual cycle progression comes from studies that demonstrated massive alterations in temporal transcription profiles upon treatment of parasites with histone deacetylase or histone acetyltransferase inhibitors [74], [75]. Together, these results demonstrate that the chromatin landscape is dynamically modified and important for the regulation of progression through the asexual blood cycle.

**Figure 2:**
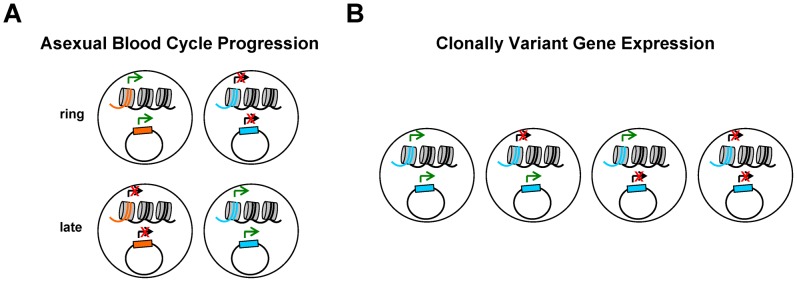
Schematic representation of transcriptional activity of episomal promoters in transfection experiments. The string of nucleosomes represents chromosomal genes, whereas the circle represents an episome. Colored lines or boxes represent a promoter. (**A**) Promoter sequences placed in an episome recapitulate correct temporal expression. Orange and blue represent a ring stage–specific promoter and a late stage–specific promoter, respectively. (**B**) The transcriptional status of clonally variant promoters often does not coincide with the state of the endogeneous promoter. Possible states for endogenous and episomal promoters are represented. See Box 1 for details.

How these chromatin modifications are regulated remains unclear, but it is unlikely that they transmit information for cycle progression from one generation of asexual parasites to the next (Figure 1B). Progression along the asexual blood cycle does not involve a choice: under the same conditions, parasites with the same genome sequence will regulate cycle progression identically, following a hard-wired program. Hence, we consider that the inherited information to control asexual cycle progression in *Plasmodium* lies within the DNA sequence, both in regulatory *cis*-acting modules and in the genes encoding transcription factor networks. This hypothesis is supported by the observation that promoters on transfected plasmids retain correct stage-specific patterns of activity [36], [76]–[78], as these plasmids are devoid of chromatin modifications prior to transfection (Box 1 and Figure 2). Furthermore, chromatin modifications associated with intra-erythrocytic development do not persist through the full asexual blood cycle, as would be expected for true epigenetic marks. Initially, the paucity of genes encoding discernable sequence-specific transcription factors in *P. falciparum* relative to its genome size [79], [80] gave reason to speculate that the chromatin landscape may primarily orchestrate temporal transcriptional changes in *Plasmodium* parasites. However, the identification of the ApiAP2 family of transcription factors, which conceivably has sufficient members to coordinate cascades of gene expression [81], [82], has somewhat rebutted this view. Nevertheless, chromatin modifications play an important role in asexual blood cycle progression, but they are more likely to represent downstream events that facilitate execution of the information contained in the DNA sequence, rather than transmitting the information themselves. Some histone PTMs that correlate with active transcription are a consequence of the process rather than responsible for it. Indeed, work in model eukaryotes has revealed that the enzymes responsible for H3K4 or H3K36 methylation are recruited to the transcriptional start sites or the entire transcribed region, respectively, by functionally distinct RNA polII isoforms [5], [7], [67]. In turn, once established, these histone PTMs facilitate the action of RNA pol II through the chromatin template. An analogous situation is observed for some histone acetylation marks that are a consequence of *trans*-activator binding [5].

**Box 1. What Do Transfection Experiments Tell Us about Epigenetic Transmission of Information in Malaria Parasites?**

In transfection experiments, naked plasmid DNA produced in bacteria is introduced into parasite nuclei, and chromatin is assembled de novo on the episome during S phase. In transient transfection experiments, promoter activity is analyzed in the first or second cycle after transfection, whereas in stable transfection experiments episomes are maintained for many generations. In both types of experiments, the episomes do not carry epigenetic information from before transfection. However, temporal regulation of promoter activity is maintained in plasmid promoters, recapitulating the correct stage-specificity of the endogenous promoter (Figure 2A) [36], [76]–[78]. This indicates that the information for temporal regulation is encoded in the promoter sequence, possibly in DNA motifs that are recognized by stage-specific transcription factors. In contrast, the active or silenced state of episomal promoters of clonally variant genes often does not coincide with the state of the endogenous variant gene. Promoters of *var* genes are silenced by default in the majority of parasites, regardless of the state of the endogenous promoter, at least when coupled to a second transcriptional unit such as the *var* intron [26], [43], [46]. Promoters of other clonally variant genes are either active by default [36], [78] or clonally variant but independent of the state of the endogenous gene (VMC and AC, unpublished data) (Figure 2B). These results indicate that promoter sequences, in coordination with *trans*-acting factors, dictate temporal regulation, whereas chromatin conformation, which does not necessarily coincide between episomal and endogenous promoters, regulates CVGE.

### Genome Indexing

The demarcation of different functional elements within the genome is another process in which chromatin modifications are involved, including histone PTMs, histone variants, and specific nucleosome positioning. In model eukaryotes, elements of the genome with particular functions in regulating transcription (such as promoters, coding regions, enhancers, or insulators) show specific chromatin features [66], [83]–[85]. Similarly, in *P. falciparum* the H3K4me3 and H3K9ac marks as well as the histone variant H2A.Z define euchromatic intergenic regions [72]. However, while in other eukaryotes H3K4me3 or H2A.Z are mainly found in nucleosomes around transcriptional start sites, in *P. falciparum* these modifications typically occupy the full intergenic region [72]. The observation that the primary sequence of episomal *Plasmodium* promoters recapitulates the function of the endogenous elements [77] (Box 1) suggests that the distribution of chromatin modifications in these functional regions is dictated by the underlying DNA sequence. Chromosomal landmark regions such as centromeres or telomeres are also characterized by specific chromatin landscapes. In many eukaryotes, centromeric DNA sequences are neither necessary nor sufficient to determine centromere activity, which has led to the idea that centromere identity is determined epigenetically by specialized chromatin structures. These structures include replacement of canonical histone H3 by centromeric CenH3, and heterochromatin assembly in pericentromeric regions [86]. Thus far it is clear that *P. falciparum* chromosome ends are demarcated by H3K9me3/HP1, similar to other eukaryotes, but the centromeres of this parasite are unusual because they are characterized by a specific sequence signature and contain CenH3 but not pericentric heterochromatin [29], [87]–[89]. Further investigations are needed to determine the relative contribution of DNA sequence and epigenetic elements in specifying centromere position in *P. falciparum*.

### Adaptive Transcriptional Responses

Yet another process where chromatin modifications play an important role in many eukaryotes is adaptation via directed transcriptional responses. When an external signal or condition is sensed, resulting in an adaptive transcriptional response, it often involves chromatin alterations [3], [27], [90]. These chromatin alterations carry epigenetic information only if they are stably maintained after the external event disappears [4]. Otherwise, if two genetically identical cells display chromatin and transcriptional differences only when they experience different external conditions, the differences are dictated by the environment and the genetic program that enables the cell to respond to it, for instance through genes encoding sensors and signal transducers (Figure 1C). In the case of malaria parasites, there is currently no evidence for directed transcriptional responses linked to chromatin changes that are maintained after the triggering event disappears. In fact, whether or not *P. falciparum* is able to sense the environment and produce adaptive transcriptional responses remains controversial [91]–[94]. The presence of sirtuins in *P. falciparum* [44], [45], [48] suggests that this parasite may be able to respond to environmental stress, as their orthologs in other eukaryotes are involved in the response to stresses such as oxidative stress or calorie restriction [95], [96]. However, such a possible role for malaria sirtuins awaits experimental demonstration.

## Concluding Remarks

Most of this opinion article focused on the *P. falciparum* asexual blood cycle because chromatin modifications and CVGE have only been characterized in some detail during this part of the life cycle. Likewise, little is known about epigenetics and chromatin modifications in other *Plasmodium* species. Chromatin changes undoubtedly play a key role in multiple aspects of parasite biology. However, to date CVGE has emerged as the only regulatory process where there is actual flow of epigenetic information from one generation to the next through heritable chromatin modifications. Importantly, CVGE is also the only known chromatin-based process for which alternative options for the parasite exist, i.e., to express or not to express a clonally variant gene. Chromatin modifications confer the ability to “remember” the option chosen in the previous generation. In contrast, in other processes such as asexual blood cycle progression, the recruitment of chromatin modifying enzymes and the resulting nucleosome modifications are downstream regulatory events that do not transmit differential information between genetically identical parasites. There are no alternative options for the parasite, and the single possible option is dictated by the genetic program, which makes epigenetic transmission of information unnecessary. This situation is different from development in multicellular eukaryotes, where in different cell types the same genome will translate into different stable patterns of gene expression that must be “remembered” over multiple generations.

An important consideration is that some of the same chromatin modifications that we consider unlikely to carry epigenetic information in processes such as stage-specific transcription or genome indexing (H2A.Z, H3K4me3, or H3K9ac) are also used in the epigenetic inheritance of CVGE and are thus involved in both types of processes [33], [50], [51]. There are examples of analogous situations in model eukaryotes: for instance, H3K4me3 is in some cases a consequence rather than a cause of transcription initiation [5], [7], but it is likely to play a role as an epigenetic mark in the context of embryonic bivalent chromatin domains [97].

Although research on *Plasmodium* epigenetics has gained momentum in recent years, our knowledge in this field is still very limited and much remains to be discovered. We still do not understand the actual mechanisms underlying the establishment and maintenance of alternative states of transcription in CVGE. Furthermore, epigenetic inheritance and/or CVGE are likely to play an important role in other life cycle stages where alternative decisions are made, such as gametocyte conversion or *P. vivax* hypnozoite formation.
